# Evaluation of manual and robot-assisted electrode placement in malformed cochleae using imaging-based patient specific 3D models

**DOI:** 10.1007/s00405-026-10611-3

**Published:** 2026-09-19

**Authors:** Fiona Anna Molnár, Mária Matuz, Zoltán Tobiás, Zsolt Geretovszky, Zsófia Bere, Haadi Mollabux, Roland Nagy, László Rovó, Ádám Perenyi

**Affiliations:** 1 Department of Otorhinolaryngology, Head and Neck Surgery, University of Szeged, Szeged, Hungary; 2 Institute of Clinical Pharmacy, Faculty of Pharmacy, University of Szeged, Szikra Street 8, Szeged, Hungary; 3 Institute of Clinical Pharmacy, Albert Szent-Györgyi Health Center, University of Szeged, Semmelweis Street 6-8, Szeged, Hungary; 4 3D Centre, Interdisciplinary Research, Development, and Innovation Excellence Center, University of Szeged, Szeged, Hungary; 5 Department of Radiology, Faculty of Medicine, University of Szeged, Szeged, Hungary

**Keywords:** Cochlear Implantation, 3D printing, Virtual models, Cochlea malformations, Medical education, Robotic Surgery.

## Abstract

**Purpose:**

Cochlear implantation in congenital inner ear malformations can be technically challenging due to the atypical anatomy which can limit electrode selection, trajectory, and insertion depth. Although high-resolution CT/MRI and planning tools (e.g., OTOPLAN^®^) support preoperative assessment, severe malformations may require additional validation. We evaluated whether robot-assisted insertion improves insertion performance in malformed cochleae using patient-specific 3D models.

**Methods:**

High-resolution CT and/or MRI from 8 paediatric candidates were used to reconstruct and 3D print 16 cochleae (malformed cochleae plus one anatomically normal control with eighth nerve aplasia, as applicable). Four electrode arrays (MED-EL Medium, MED-EL Compressed, MED-EL Flex24, and Cochlear Straight) were inserted by an expert surgeon (P1), a novice surgeon (P2), and a robotic system (RobOtol^®^, R1). Outcomes were insertion completeness (complete/incomplete) and ease (easy/hard). Data were analysed using Firth’s penalized logistic regression and classification tree analysis. Preoperative prediction by P1 alone versus P1 + OTOPLAN^®^ was compared using standard classification metrics.

**Results:**

Electrode type and cochlear anatomy were the primary determinants of insertion outcomes. Electrode arrays with shorter active lengths (MED-EL Compressed) demonstrated higher insertion completeness in anatomically restricted malformed cochleae, whereas Flex24 and Cochlear Straight showed reduced completeness in restrictive hypoplastic cochleae. Operator identity, including robotic assistance, had minimal impact; ease was generally high. OTOPLAN^®^ assisted planning improved prediction performance and reduced false-negative assessments.

**Conclusion:**

In malformed cochleae, insertion completeness was influenced primarily by cochlear morphology and electrode characteristics within the outcome measures assessed. Patient-specific 3D models provide a useful platform for electrode–anatomy compatibility assessment and preoperative planning; however, clinical validation incorporating tonotopic coverage, audiological outcomes, and objective robotic performance metrics is warranted.

**Supplementary Information:**

The online version contains supplementary material available at https://doi.org/10.1007/s00405-026-10611-3.

## Introduction

Cochlear implantation (CI) is a standard treatment for severe to profound sensorineural hearing loss, providing significant auditory rehabilitation and improved quality of life. However, implantation in patients with congenital cochlear malformations, such as cochlear hypoplasia or anomalies associated with rare syndromes, presents unique surgical challenges. Aberrant cochlear anatomy can complicate electrode selection, insertion, and placement, thereby increasing the risk of electrode displacement, cerebrospinal fluid (CSF) gusher, and postoperative complications [1, 2]. Outcomes in patients with inner ear malformations (IEM), such as incomplete partition type III (IP-III), have historically been reported as poorer compared with those in paediatric CI recipients with normal inner ear anatomy [1]. For this reason, many centres remain cautious about implanting IEM patients due to uncertainty regarding auditory outcomes and the increased risk of intraoperative challenges, including perilymph leak, CSF gusher, facial nerve injury, and electrode migration [2].

Radiological assessment is therefore essential in the work-up of CI candidates with severe sensorineural hearing loss (SNHL), especially those suspected of harbouring inner ear anomalies. High-resolution computed tomography (CT) and magnetic resonance imaging (MRI) are indispensable for diagnosing and classifying malformations such as IP-III and cochlear hypoplasia, as well as for delineating cochlear duct patency, cochlear nerve presence, and the spatial relationship to adjacent neurovascular structures [3, 4]. Sennaroğlu et al., 2017 provided the foundational classification of IEMs based on temporal bone CT and MRI, which remains the reference for modern diagnostic practice [2, 5]. Accurate imaging-based classification is critical not only for confirming the diagnosis but also for guiding surgical approach and predicting postoperative auditory outcomes.

Preoperative imaging has evolved beyond diagnostic characterization toward quantitative and functional planning. Measurement of cochlear dimensions - such as basal turn diameter, cochlear height, and cochlear duct length (CDL) - is now an integral part of CI preparation, as these variables inform electrode selection and insertion depth [6]. The dedicated surgical planning software OTOPLAN^®^ allows for three-dimensional (3D) visualization of the cochlear anatomy and enables virtual simulation of different electrode arrays. It facilitates individualized frequency mapping for each electrode contact, allowing surgeons to anticipate insertion angles and optimize the postoperative frequency allocation. These tools are particularly valuable in malformed cochleae, where atypical geometry complicates manual estimation of cochlear duct length and trajectory. However, even advanced image-based simulation can be limited by the complexity of malformed cochlear architecture, necessitating further tactile and spatial validation through physical modelling and a manual trial prior to surgery.

Recent advancements in 3D imaging and 3D printing technology have introduced new opportunities for patient-specific surgical planning. Using high-resolution CT and MRI data, platforms such as 3D Slicer enable accurate 3D reconstruction of the bony labyrinth and ossicular chain, which can subsequently be materialized via 3D printing in biocompatible resin. While previous applications of these techniques have focused on conditions such as cholesteatoma with fistulas and complex labyrinthine fistulas, they have seldom been applied to congenital inner ear malformations. Temporal bone and cochlear anomalies occur in approximately 10% of patients with congenital severe-to-profound SNHL [7]. The use of 3D-printed, patient-specific models has demonstrated high anatomical accuracy and has been increasingly adopted for surgical simulation and preoperative training in challenging otologic and neurosurgical cases such as in patients with complex labyrinthine fistulas and fistulas in cholesteatoma patients [7]. Despite these developments, the extent to which 3D-printed, patient-specific models can meaningfully enhance electrode choice in patients with complex inner ear malformations undergoing cochlear implantation remains largely unexplored.

Parallel to imaging and modelling innovations, the integration of robotic assistance into otologic surgery represents another major technological advancement. Robot-assisted cochlear implantation (RACI) has been developed to enhance precision, stability, and reproducibility in electrode insertion. Robotic platforms - including RobOtol^®^, OTODRIVE^®^, IotaSoft^®^, and other hydrodrive and roller-wheel mechanisms - offer controlled, steady insertion along pre-defined trajectories, reducing the likelihood of insertion trauma [8]. These systems help address key challenges faced by surgeons, including the difficulty of navigating complex anatomy, the need for precise electrode trajectory control, and intraoperative variability. They offer reduced insertion forces, lower intracochlear pressure, and improved placement accuracy [8]. Additionally, robotics have been applied in related procedures such as mastoidectomy, cochleostomy, and direct cochlear access, demonstrating increasing procedural reliability.

In malformed cochleae, where landmarks may be distorted and insertion resistance unpredictable, robot-assisted insertion could provide critical consistency and minimize acute intracochlear trauma associated with manual electrode advancement. Reducing insertion-related trauma is clinically meaningful: despite improvements in electrode design and surgical technique, up to 50% of hearing preservation CI recipients experience a postoperative hearing threshold shift greater than 10 dB, and approximately 10–50% lose functional residual hearing entirely [9]. Robotic assistance has also been associated with reduced electrode impedances, a surrogate biomarker for decreased cochlear fibrosis and better long-term preservation of residual hearing [9]. Despite the growing use of robotic systems and 3D planning tools, systematic assessment of robot-assisted electrode insertion in anatomically malformed cochleae remains unexplored. Integrated evaluations using 3D reconstruction, physical models, and robotic insertion across electrode types and operators remain scarce.

## Materials and methods

**Fig. 1:**
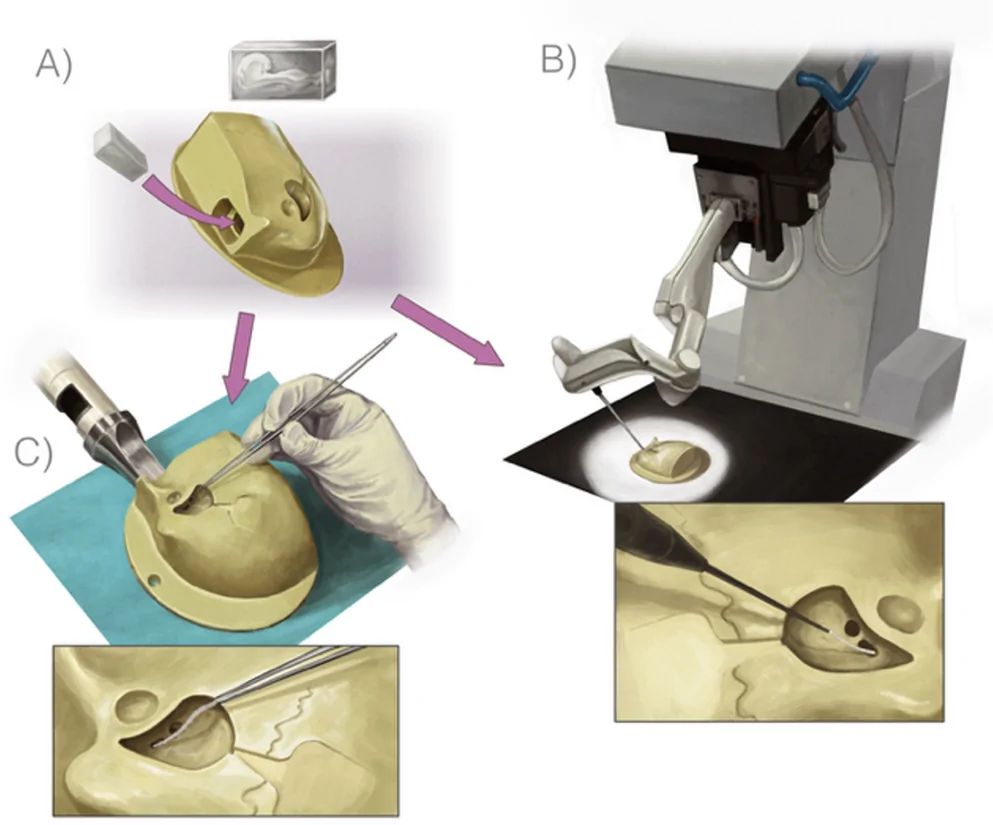
Manual and robotic electrode insertion workflows using 3D-printed cochlear models. Schematic overview of the experimental setup comparing manual and robot-assisted electrode insertion techniques. (**A**) Patient-specific 3D-printed cochlear model generated from CT/MRI data following manual segmentation and stereolithographic fabrication. (**B**) Robotic insertion workflow using the RobOtol system, showing robot-guided positioning and automated advancement of the electrode array into the cochlear model. (**C**) Manual electrode insertion performed using standard microsurgical instruments. Insets illustrate close-up views of electrode positioning and progression within the cochlear lumen. These models were used to evaluate feasibility of insertion, trajectory control, and performance of manual versus robotic techniques across varied inner ear anatomies

In the present study, we employed high-resolution CT and MRI imaging to generate patient-specific 3D reconstructions of 16 cochleae (malformed cochleae plus one anatomically normal control with 8th cranial nerve aplasia) from 8 patients. Using the resulting 3D-printed models, we compared four commercially available electrode arrays and evaluated the completeness and ease of insertion under manual and robotic conditions (Fig. 1).

### Patient selection

This study included patients of any age and gender who have any form of cochlea malformation identified prior to cochlear implant surgery for severe sensorineural hearing loss. Patients were included over a three-year period up to May 2025 (Table 1). Patients with significant imaging artifacts, previous inner ear surgery were excluded to ensure the segmentation accuracy. High-resolution computed tomography (CT) and MRI scans were obtained and anonymised to protect patient confidentiality, in accordance with institutional and national data protection regulations and ethical requirements. All procedures were approved in 2024 by the XXXXX committee (Approval number: XXXXX).

**Table 1:**
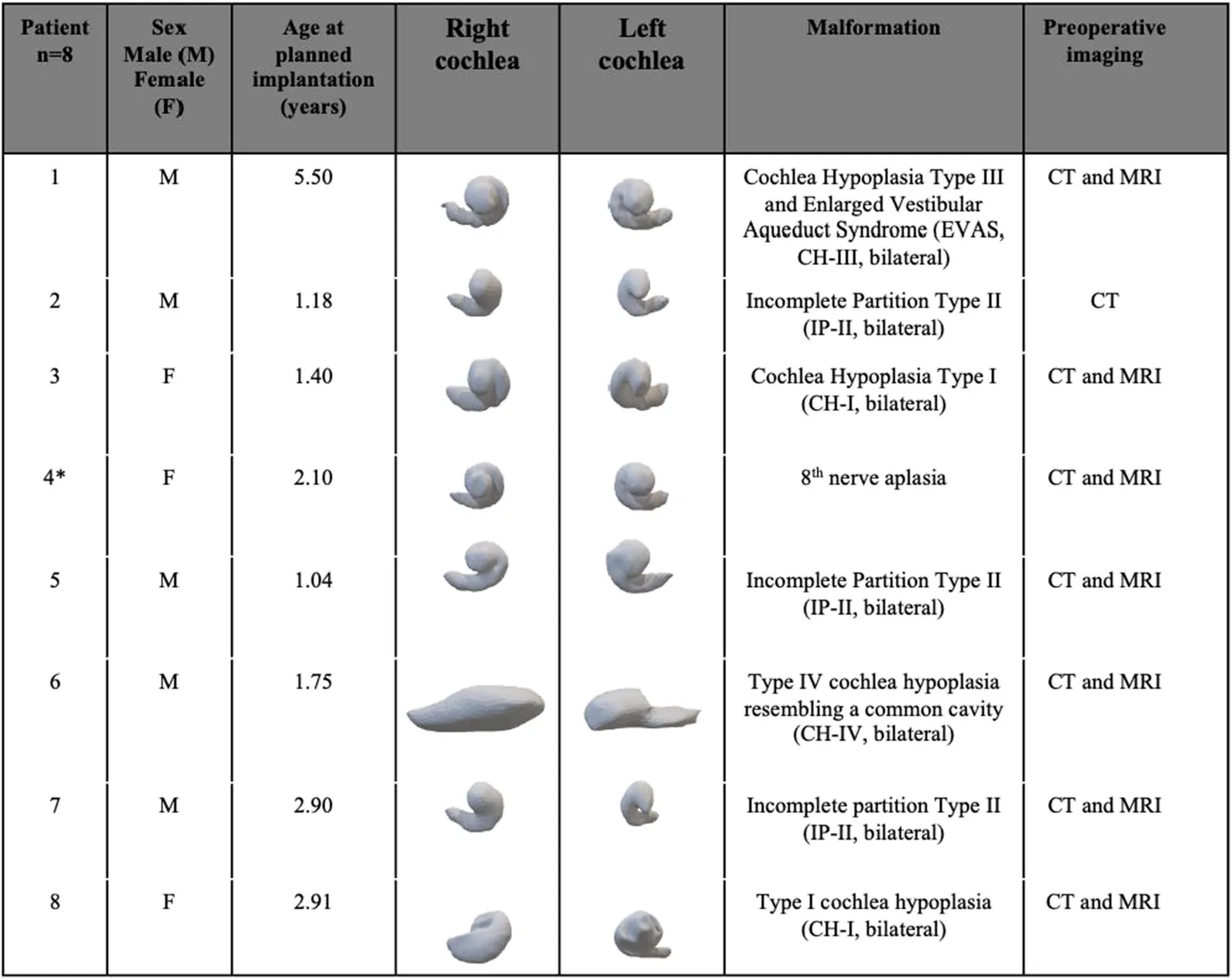
Clinical characteristics of the cochlear implant recipients included in the study. A summary of patient demographics, type of inner ear malformation, laterality of malformation, and preoperative imaging used. Malformation types follow established radiological classifications (e.g., incomplete partition type II, cochlear hypoplasia). Patient 4 had a structurally normal cochlea and therefore served as a control. Preoperative imaging consisted of high-resolution computed tomography (CT) and/or magnetic resonance imaging (MRI) to confirm anatomical suitability for cochlear implantation. For each patient, both the right and left cochleae were reconstructed, segmented, and 3D printed (*n* = 16) to demonstrate the morphology and shape of malformations present in each individual. These 3D models are intended for qualitative visualization only and are not to scale relative to actual cochlear dimensions

The image-based 3D modelling and printing was applied to a cohort of eight patients (totalling 16 cochleae, left and right side, Table 1). The mean age of the patients was 2.35 ± 1.46 years and the cohort included a broad spectrum of congenital inner ear malformations, including CH-III and EVAS (*n* = 1), IP type II (*n* = 3), cochlear hypoplasia of various subtypes (*n* = 3), and a control patient with a normal cochlea and 8th nerve aplasia (*n* = 1, totalling two cochleae – left and right side, Table 2). In total three P1, three P2, and three R1 insertions were performed per patient per electrode.

### 3D segmentation and 3D model generation

Cochlear segmentation was performed using the 3D Slicer image computing platform (version 5.8.1) following a workflow as we described previously [10]. Due to the varied morphology of the malformed cochleae, segmentation was conducted manually for each case, focusing on the cochlear lumen, vestibule, and semicircular canals [10]. Where available, MRI data were fused with CT to provide enhanced soft-tissue contrast. Each segmented structure was assigned a specific label and exported as a 3D model. The resulting models were converted to Stereolithography (STL) format and printed using a 3D Systems ProJet 6000 HD stereolithography printer with VisiJet ClearVue biocompatible resin (Fig. 1).

### Electrode types and virtual planning

Each of the 16 cochlear models were inserted with four different commercial electrode arrays: MED-EL Medium, MED-EL Compressed, MED-EL Flex 24 (Innsbruck, Austria) and Cochlear™ Slim Straight electrode (Sydney, Australia) arrays (Table 2) [11]. Selection criteria for the optimal electrode included mechanical fit, insertion feasibility, and proximity to presumed neural targets in cases of cystic or distorted cochlear architecture.

**Table 2:** Characteristics of cochlear implant electrode arrays evaluated in this study

Electrode Array	Manufacturer	Active Length (mm)	Stimulation Channels	Intended application
MED-EL Standard	MED-EL	~ 26.4	12 (12 pairs)	Standard-length lateral wall electrode for normal cochlear anatomy
MED-EL Compressed	MED-EL	~ 12.1	12 (12 pairs)	Short lateral wall electrode for malformed or hypoplastic cochleae
MED-EL Flex 24	MED-EL	~ 20.9	12 (5 single, 7 pairs)	Flexible lateral wall electrode for atraumatic insertion and hearing preservation
Cochlear Slim Straight	Cochlear	~ 19.1	22 (22 single)	Lateral wall electrode with half-banded contacts oriented toward the modiolus

### Prediction of electrode insertion based on preoperative imaging using 3D modelling software

Prior to physical insertions, an experienced cochlear implant surgeon and radiologist (P1) performed a preoperative assessment of each cochlea using anonymized patient imaging (CT or MRI). In the first approach, electrode selection was based solely on this patient imaging and P1’s evaluation. In the second approach, electrode planning for MED-EL electrodes incorporated both P1’s assessment and OTOPLAN^®^ software (MED-EL, Innsbruck, Austria; Cascination AG, Bern, Switzerland), following the standard departmental protocol for cochlear implant preoperative planning. For both approaches, the evaluation included measurement of cochlear dimensions and identification of anatomical variations to determine the most appropriate electrode type for each cochlea. This methodology ensured that electrode choice reflected individualized cochlear anatomy while maintaining consistency with clinical practice.

### Robotic-assisted planning and insertion trials

Robot-assisted electrode insertions were carried out using the RobOtol (Collin Medical, Bagneux, France) system. Insertion orientation was manually determined based on the patient-specific 3D models and imaging findings (Fig. 1). Each electrode insertion was then physically tested on the 3D-printed models by an Expert Otolaryngologist (P1), a Novice Otolaryngologist (P2, with only prior observational experience), and a robotic system (R1) under the supervision of P1.Electrode insertion was performed using the RobOtol robotic system at a constant speed of 0.3 mm/s the maximum insertion force or intracochlear pressure was not directly measured during the procedure. However, based on published experimental data at comparable insertion speeds, the maximal insertion force at 0.3 mm/s is estimated to lie in the range of approximately 0.036 N as reported by Majdani et al. (2010) to 0.044 ± Aebischer et al. (2022) [12, 13]. These values provide an indicative estimate of the insertion forces associated with the insertion speed used in the present study. These estimates should be interpreted with caution, as they are derived from in vitro studies using synthetic cochlea models, which may not fully replicate the biomechanical properties of the human cochlea. Following each insertion, outcomes were assessed qualitatively and categorized using predefined criteria. Insertion completeness was classified as complete (score = 1) when the electrode array was fully inserted to the intended depth without premature termination, or incomplete (score = 0) otherwise. Insertion difficulty was classified as easy (score = 1) when insertion proceeded smoothly without the need for excessive force, repeated repositioning, or interruption, and hard (score = 0) when increased resistance, repeated adjustments, or procedural difficulty was encountered during advancement of the electrode array.

This scoring enabled comparative evaluation of performance across operators (P1, P2 or R1) and electrode array types and provided insight into the usability of robot-assisted techniques in anatomically complex cases. To minimise subjectivity, predefined criteria were applied consistently across all insertions, including the need for increased insertion force, repeated repositioning, or interruption of advancement. Nevertheless, this metric retains an inherent degree of operator-dependent interpretation.

### Statistical analysis

Binary outcomes (*completeness* and *ease*) were analysed using Firth’s bias-reduced logistic regression. This method was selected because several operator–electrode–patient combinations exhibited separation, where standard maximum-likelihood logistic regression yields infinite or unstable parameter estimates. The bias-reduced approach provides finite, more reliable estimates and valid standard errors under these conditions.

The models included the fixed effects operator, electrode type, side (left/right), and the operator–electrode interaction. Patient ID (PID) was incorporated as a fixed effect to account for patient-level heterogeneity and to avoid instability associated with estimating random effects in a small number of clusters. PID effects were included only for adjustment and were not interpreted clinically.

Effects were summarized as odds ratios (OR) with 95% Wald confidence intervals derived from the bias-reduced estimates. All analyses were performed using R with the *brglm2* package. To analyse the binary outcome variables insertion completeness and ease of insertion, a classification tree analysis was performed using IBM SPSS Statistics (version 31) with the Classification and Regression Tree (CRT) algorithm. The model included patient identifier (PID), electrode array type, operator (P1, P2, R1), and side of implantation (left/right) as explanatory variables.

The predictive performance of preoperative electrode selection based on clinician assessment alone (P1) and clinician assessment combined with OTOPLAN^®^ software (P1 + OP) was evaluated by comparing binary predicted outcomes with observed insertion success in physical model trials. Classification performance was assessed using accuracy, sensitivity, specificity, PPV, NPV, F1 score, balanced accuracy, MCC, and Cohen’s kappa, derived from contingency tables of predicted versus measured outcomes.

## Results

### Insertion completeness

A multivariable logistic regression model was used to evaluate factors associated with complete electrode insertion, with complete insertion as the dependent variable (Table 3). Operator (reference: P1), electrode type (reference: MED-EL Compressed), and side of implantation (reference: left ear) were included as fixed effects, with interaction terms between operator and electrode type to assess potential effect modification; patient identity was included to account for subject-level variability. Odds ratios greater than 1 indicate higher odds of complete insertion, whereas odds ratios less than 1 indicate lower odds, with statistical significance defined as *p* < 0.05.

**Table 3:** Multivariable logistic regression analysis of factors associated with complete electrode insertion.

	beta	SE	z	*p*	OR	CI_low	CI_high
**(Intercept)**	**2.421**	**0.772**	**3.138**	**0.002**	**11.260**	**2.482**	**51.094**
OperatorP2	0.000	0.885	0.000	1.000	1.000	0.176	5.669
OperatorR1	2.205	1.592	1.385	0.166	9.072	0.401	205.349
Electrode array 2 Cochlear™ Slim Straight	**−2.496**	**0.818**	**−3.053**	**0.002**	**0.082**	**0.017**	**0.409**
**Electrode array 3** **MED-EL Flex 24**	**−5.353**	**0.925**	**−5.790**	**0.000**	**0.005**	**0.001**	**0.029**
**Electrode array 4** **MED-EL Medium**	**−3.256**	**0.850**	**−3.832**	**0.000**	**0.039**	**0.007**	**0.204**
Side (Right)	−0.491	0.326	−1.506	0.132	0.612	0.323	1.160
OperatorP2: Electrode array 2 Cochlear™ Slim Straight	−2.265	1.176	−1.927	0.054	0.104	0.010	1.040
**OperatorR1: Electrode array 2 Cochlear™ Slim Straight**	**−5.357**	**1.781**	**−3.007**	**0.003**	**0.005**	**0.000**	**0.155**
OperatorP2: Electrode array 3 Flex 24	0.592	1.163	0.509	0.611	1.808	0.185	17.681
OperatorR1: Electrode array 3 Flex 24	−1.911	1.760	−1.086	0.277	0.148	0.005	4.654
OperatorP2: Electrode 4 Med-El Med	0.000	1.151	0.000	1.000	1.000	0.105	9.545
**OperatorR1: Electrode array 4 MED-EL Medium**	**−3.710**	**1.770**	**−2.096**	**0.036**	**0.024**	**0.001**	**0.786**

Multivariable logistic regression results for complete electrode insertion. Regression coefficients (β), standard errors (SE), Wald z-statistics, p-values (p), odds ratios (OR), and 95% confidence intervals (CI) are shown. Effects of operator, electrode type, side of implantation, and operator–electrode interaction terms are reported relative to their respective reference categories. The statistically significant differences are highlighted in bold

No significant differences were observed between operators, as neither the novice operator (P2; OR = 1.00, 95% CI 0.18–5.67, *p* = 1.00) nor the robotic system (R1; OR = 9.07, 95% CI 0.40–205.35, *p* = 0.166) differed significantly from the reference operator (P1).

In contrast, electrode design showed a pronounced effect: compared with the MED-EL Compressed array, the odds of complete insertion were significantly lower for the Cochlear™ Slim Straight (OR = 0.082, 95% CI 0.018–0.41, *p* = 0.0023), Flex 24 (OR = 0.0047, 95% CI 0.0007–0.028, *p* < 10⁻⁸), and MED-EL Medium electrodes (OR = 0.038, 95% CI 0.008–0.18, *p* < 0.001, Table 3). A significant operator–electrode interaction was observed for the Cochlear Slim Straight electrode with the robotic system (OR = 0.005, 95% CI 0–0.16, *p* = 0.003), along with a borderline interaction for P2 (*p* = 0.054), indicating that this electrode design was particularly unfavourable for certain operators, whereas no such effects were observed for other arrays. Side of implantation was not significantly associated with insertion completeness (*p* = 0.13). Patient-specific effects were substantial, with marked variability in the odds of complete insertion across malformed cochleae, highlighting the dominant influence of individual cochlear anatomy on insertion outcome.

Insertion completeness was strongly influenced by cochlear size, particularly in hypoplastic cochleae identifiable on preoperative CT and/or MRI. In these anatomically restricted cases, complete insertion is feasible only when electrode length is compatible with the available cochlear lumen. Where cochlear duct length could be estimated preoperatively, electrodes exceeding the available duct length were considered unlikely to achieve complete insertion by definition. Accordingly, incomplete insertion in such cases should not necessarily be interpreted as a technical failure but may reflect an expected anatomical limitation due to electrode–cochlea mismatch. Therefore, completeness outcomes were interpreted in the context of whether, based on preoperative estimation or measurement, the selected electrode had a realistic potential to achieve complete insertion.

### Ease of electrode insertion

Multivariable logistic regression analysis demonstrated that electrode array type and side of implantation were the primary factors associated with ease of electrode insertion, whereas operator-related effects were not significant (Table 4). Compared with the reference electrode (MED-EL Compressed), the odds of easy insertion were significantly lower for the Cochlear™ Slim Straight array (OR = 0.005, 95% CI 0.001–0.05, *p* < 0.001), MED-EL Flex 24 (OR = 0.017, 95% CI 0.002–0.15, *p* < 0.001), and MED-EL Medium electrodes (OR = 0.056, 95% CI 0.007–0.47, *p* = 0.008). Right-sided implantation was also associated with significantly lower odds of easy insertion compared with the left side (OR = 0.082, 95% CI 0.034–0.20, *p* < 0.001).

**Table 4:** Multivariable logistic regression analysis of factors associated with ease of electrode insertion

	beta	SE	z	*p*	OR	CI_low	CI_high
**(Intercept)**	**5.475**	**1.071**	**5.113**	**0.000**	**238.583**	**29.258**	**1945.530**
OperatorP2	0.740	1.170	0.633	0.527	2.097	0.212	20.754
OperatorR1	−0.677	1.113	−0.608	0.543	0.508	0.057	4.502
**Electrode array 2 Cochlear™ Slim Straight**	**−5.270**	**1.159**	**−4.548**	**0.000**	**0.005**	**0.001**	**0.050**
**Electrode array 3** **MED-EL Flex 24**	**−4.078**	**1.127**	**−3.619**	**0.000**	**0.017**	**0.002**	**0.154**
**Electrode array 4** **MED-EL Medium**	**−2.886**	**1.085**	**−2.661**	**0.008**	**0.056**	**0.007**	**0.468**
**Side (Right)**	**−2.497**	**0.446**	**−5.605**	**0.000**	**0.082**	**0.034**	**0.197**
OperatorP2:electrode array 2 Cochlear™ Slim Straight	−0.740	1.445	−0.512	0.608	0.477	0.028	8.103
OperatorR1:electrode array 2 Cochlear™ Slim Straight	−0.465	1.404	−0.331	0.741	0.628	0.040	9.839
OperatorP2: electrode array 3 MED-EL Flex 24	−0.740	1.464	−0.506	0.613	0.477	0.027	8.412
OperatorR1:electrode array 3 MED-EL Flex 24	1.869	1.434	1.303	0.192	6.483	0.390	107.794
OperatorP2: electrode array 4 MED-EL Medium	−0.740	1.465	−0.505	0.613	0.477	0.027	8.422
OperatorR1: electrode array 4 MED-EL Medium	−0.515	1.418	−0.363	0.717	0.598	0.037	9.629

Regression coefficients (β), standard errors (SE), Wald z-statistics, p-values (p), odds ratios (OR), and 95% confidence intervals (CI) are shown. Effects of operator, electrode array type, side of implantation, and operator–electrode interaction terms are reported relative to their respective reference categories. Statistically significant differences are highlighted in bold

No significant main effects of operator were observed, with neither operator P2 (OR = 2.10, 95% CI 0.21–20.75, *p* = 0.527) nor operator R1 (OR = 0.51, 95% CI 0.06–4.50, *p* = 0.543) differing significantly from the reference operator (P1, Table 4). Similarly, none of the operator–electrode interaction terms reached statistical significance (all *p* > 0.19), indicating that the effect of electrode array on ease of insertion was consistent across operators (Fig. 1).

### Comparison of preoperative insertion predictions with and without software assistance

As outlined in the Methods, two preoperative planning approaches were evaluated: imaging-based electrode selection by an experienced clinician alone, and selection incorporating both clinician assessment and OTOPLAN^®^ software, which was applicable only to MED-EL electrode arrays and excluded the Cochlear Straight electrode. For the P1 OTOPLAN^®^ prediction, three electrodes (MED-EL Medium, MED-EL Compressed and MED-EL Flex24) were used for the sixteen cochleae thus, altogether 48 predictions were made. In contrast, the P1 assessment included four electrodes (Cochlear© Straight, MED-EL Medium, MED-EL Compressed and MED-EL Flex24) with the total of 64 predictions.

For the P1-only assessment, predictions were based on qualitative evaluation of preoperative CT and/or MRI images, taking into account cochlear morphology, degree of hypoplasia, available cochlear lumen, and the anticipated feasibility of complete electrode insertion without excessive resistance.

For the P1 + OTOPLAN^®^ approach, these clinician-based assessments were complemented by software-derived parameters, including cochlear duct length estimation, virtual electrode fitting, and predicted insertion depth, allowing a more quantitative evaluation of electrode–anatomy compatibility.

**Fig. 2:**
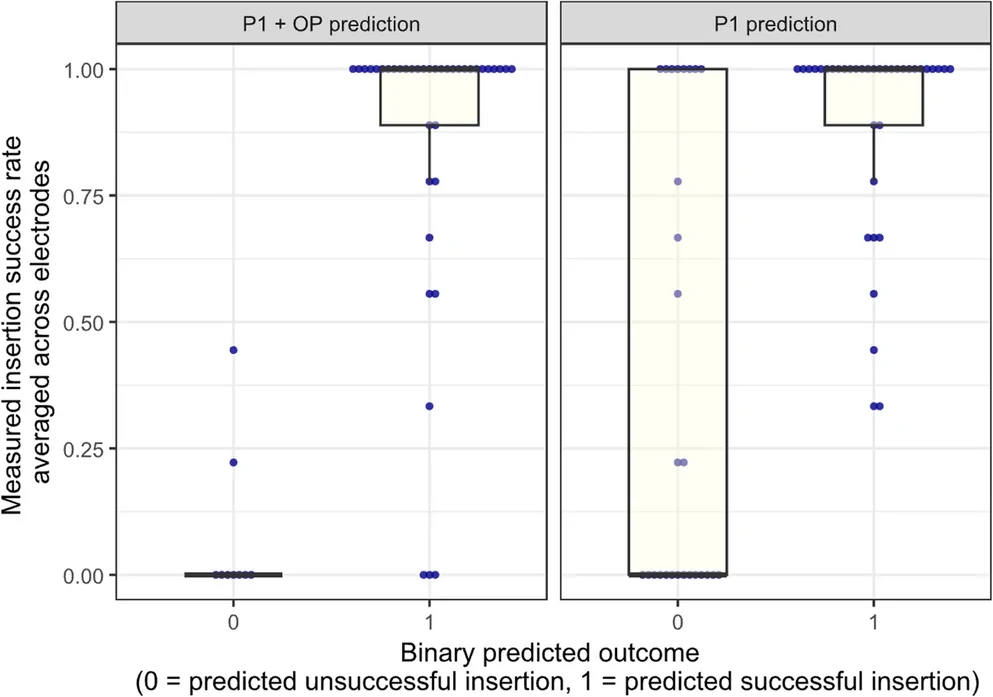
Relationship between preoperative predicted insertion outcome and measured insertion success for clinician assessment alone (P1) and clinician assessment combined with OTOPLAN^®^ software (P1 + OP). The x-axis shows the binary predicted outcome (0 = predicted unsuccessful insertion, 1 = predicted successful insertion), and the y-axis shows the measured insertion success rate averaged across electrodes. Individual points represent single predictions, with boxplots summarizing the distribution of measured outcomes for each prediction category. Incorporation of OTOPLAN^®^ resulted in clearer separation between predicted successful and unsuccessful insertions, whereas P1-only assessment showed greater overlap between prediction categories

Figure 2 illustrates the relationship between predicted insertion outcome (binary prediction: 0 (unsuccessful insertion predicted) or 1 (successful insertion predicted) and the measured insertion success rate for the two approaches. For the P1 + OTOPLAN^®^ predictions, cases predicted as unsuccessful (prediction = 0) were associated almost exclusively with very low measured success rates, clustering near zero. Conversely, cases predicted as successful (prediction = 1) showed consistently high measured success rates, with most values close to 1.0 and relatively limited dispersion. Only a small number (*n* = 2) of outliers exhibited moderate measured success despite a positive prediction, indicating strong concordance between predicted and measured outcomes.

In contrast, the P1-only predictions demonstrated substantially greater variability. While predictions of success (prediction = 1) were generally associated with high measured success rates, a wider spread of values was observed, including approximately eight observations with only moderate or low measured success. More notably, predictions of failure (prediction = 0) showed a broad distribution of measured outcomes, with a substantial number of cases (approximately *n* ≈ 13) achieving moderate to high insertion success despite being predicted as unsuccessful. This indicates that, without OTOPLAN^®^ support, negative predictions frequently included cochleae that ultimately achieved high insertion success, suggesting that P1-only assessment tended toward more cautious electrode selection.

These distributional patterns observed in Fig. 2 are quantitatively summarized in Table 5, which reports standard classification performance metrics for the two preoperative planning approaches.

**Table 5:** Performance metrics for preoperative prediction of electrode insertion success using clinician assessment alone (P1) and clinician assessment combined with OTOPLAN^®^ software (P1 + OP)

prediction_type	acc	sens	spec	ppv	npv	f1	bacc	mcc	kappa
Prediction (P1 + OP)	0.771	1.000	0.450	0.718	1.000	0.836	0.725	0.568	0.488
Prediction (P1)	0.719	0.771	0.655	0.730	0.704	0.750	0.713	0.430	0.429

Accuracy (acc), sensitivity (sens), specificity (spec), positive predictive value (PPV), negative predictive value (NPV), F1 score (f1), balanced accuracy (bacc), Matthews correlation coefficient (MCC), and Cohen’s kappa (κ) are reported. Higher values indicate better predictive performance

Consistent with the clearer separation between predicted and measured outcomes seen in the scatter plots, incorporation of OTOPLAN^®^ markedly improved sensitivity and overall agreement, achieving perfect sensitivity (1.000) and negative predictive value (1.000) with higher overall accuracy (0.771), balanced accuracy (0.725), Matthews correlation coefficient (0.568), and Cohen’s kappa (0.488), while eliminating false-negative predictions of insertion success. In contrast, P1-only assessment demonstrated lower sensitivity (0.771), reduced overall accuracy (0.719), and weaker agreement metrics (MCC = 0.430; κ = 0.429), despite a modestly higher specificity (0.655 vs. 0.450).

### Individual cochlea analysis

**Fig. 3:**
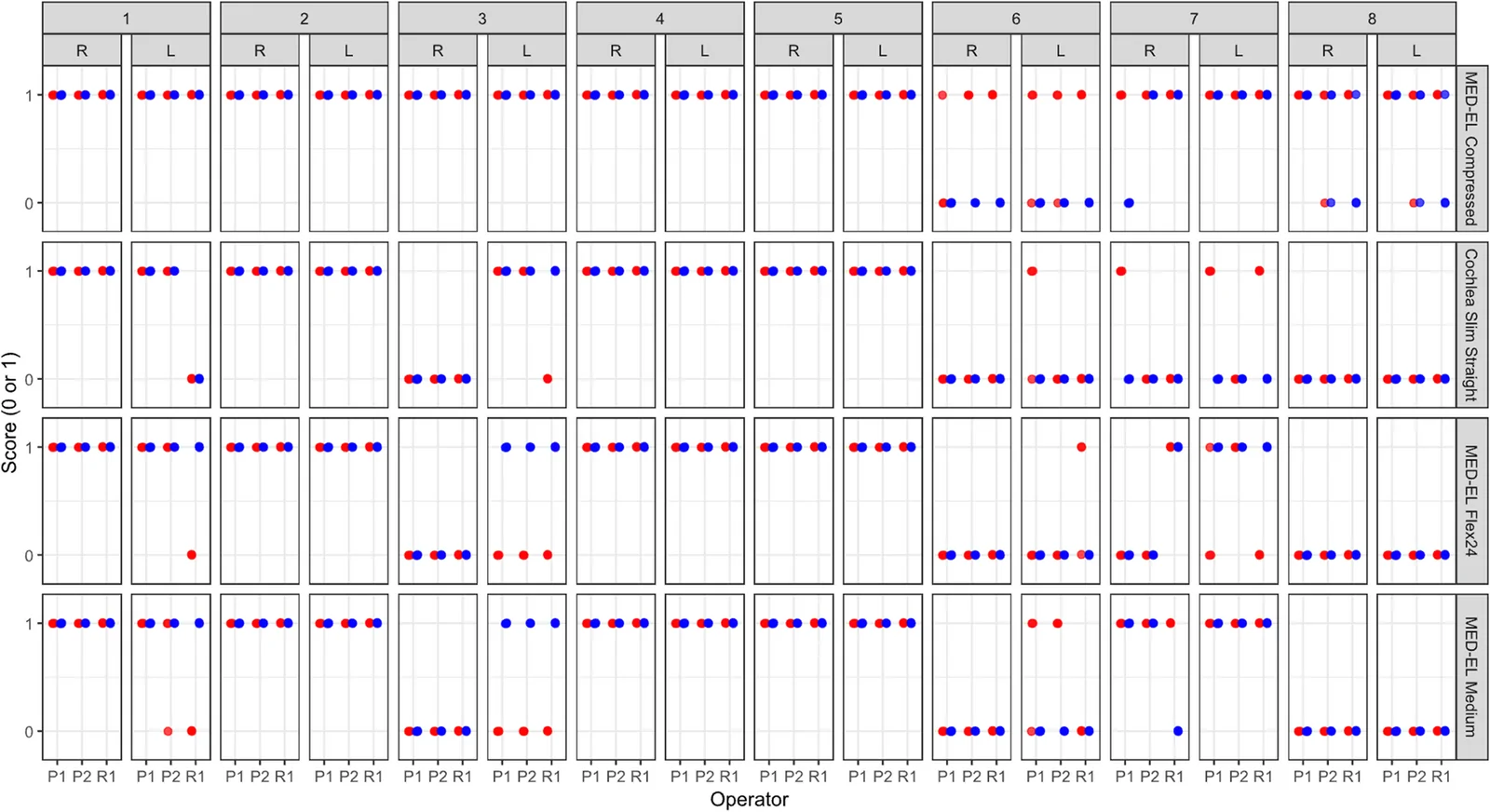
Insertion completeness and ease-of-insertion across operators, cochlear sides, and electrode types Scatter plots depict insertion completeness (red) and ease-of-insertion (blue) for each patient and cochlear side (L/R), evaluated across three operators (P1 = expert, P2 = novice, R1 = robotic system). Four electrode types (Compressed, Cochlear Straight, Flex24, and MED-EL Medium) are shown in separate rows. Scores range from 0–1, with higher values indicating successful or easy insertions. The figure demonstrates consistent ease-of-insertion across operators, while variations in completeness primarily reflect differences in electrode design and patient-specific cochlear anatomy

In the control case (Fig. 3, patient 4), complete insertion was consistently achieved across all electrode array types, operators, and both implantation sides (L and R). No incomplete insertions were observed for any array, including Cochlear™ Slim Straight, MED-EL Flex 24, MED-EL Medium, and MED-EL Compressed. In contrast to the control, substantial variability was observed across patients with malformed cochleae, with outcomes tending to be consistent within individuals (e.g. patient 6 right and left, patient 8 right and left) but differing markedly between patients (e.g. patients 1, 2, and 5 vs. 6, 7 and 8). Patients 1, 2, and 5 showed largely complete insertions across most electrode arrays, whereas patients 6, 7, and 8 exhibited recurrent incomplete insertions for specific arrays across all operators.

Across all patients and electrode arrays, operator-related effects were limited and inconsistent. No operator demonstrated systematically superior or inferior performance, and incomplete insertions were typically shared across P1, P2, and R1 within the same patient–electrode combinations. The robotic system closely mirrored human operator performance, achieving complete insertions in favourable anatomies and encountering similar limitations in anatomically constrained cases.

Side-dependent differences were observed in a subset of patients e.g. patient 6 demonstrated divergent outcomes between left and right ears for the same electrode array across operators.

When stratified by electrode type, the MED-EL Compressed array demonstrated the greatest robustness to anatomical variability, achieving complete insertion in nearly all patients (12 cochleae), including those who experienced failures with other electrode designs (e.g. patient 3 and 7). In contrast, the Cochlear™ Slim Straight array showed pronounced patient-dependent variability. In patients such as 6 (right side) and 8 (both sides), incomplete insertions occurred across all operators, whereas patients 2, 4 and 5 (both sides) consistently achieved full insertion. A similar anatomy-driven pattern was observed for the MED-EL Flex 24 electrode. Complete insertions were achieved in patients such as 2 and the control patient (patient 4), while patients 6, 7 and 8 exhibited incomplete insertions across operators and sides. The MED-EL Medium electrode exhibited intermediate performance, with successful insertions in anatomically favourable cochleae (including the control) and incomplete insertions in more constrained anatomies, such as patient 3, patient 6 and patient 8. These outcomes were consistent across operators.

### Classification tree analysis of factors influencing insertion success

**Fig. 4:**
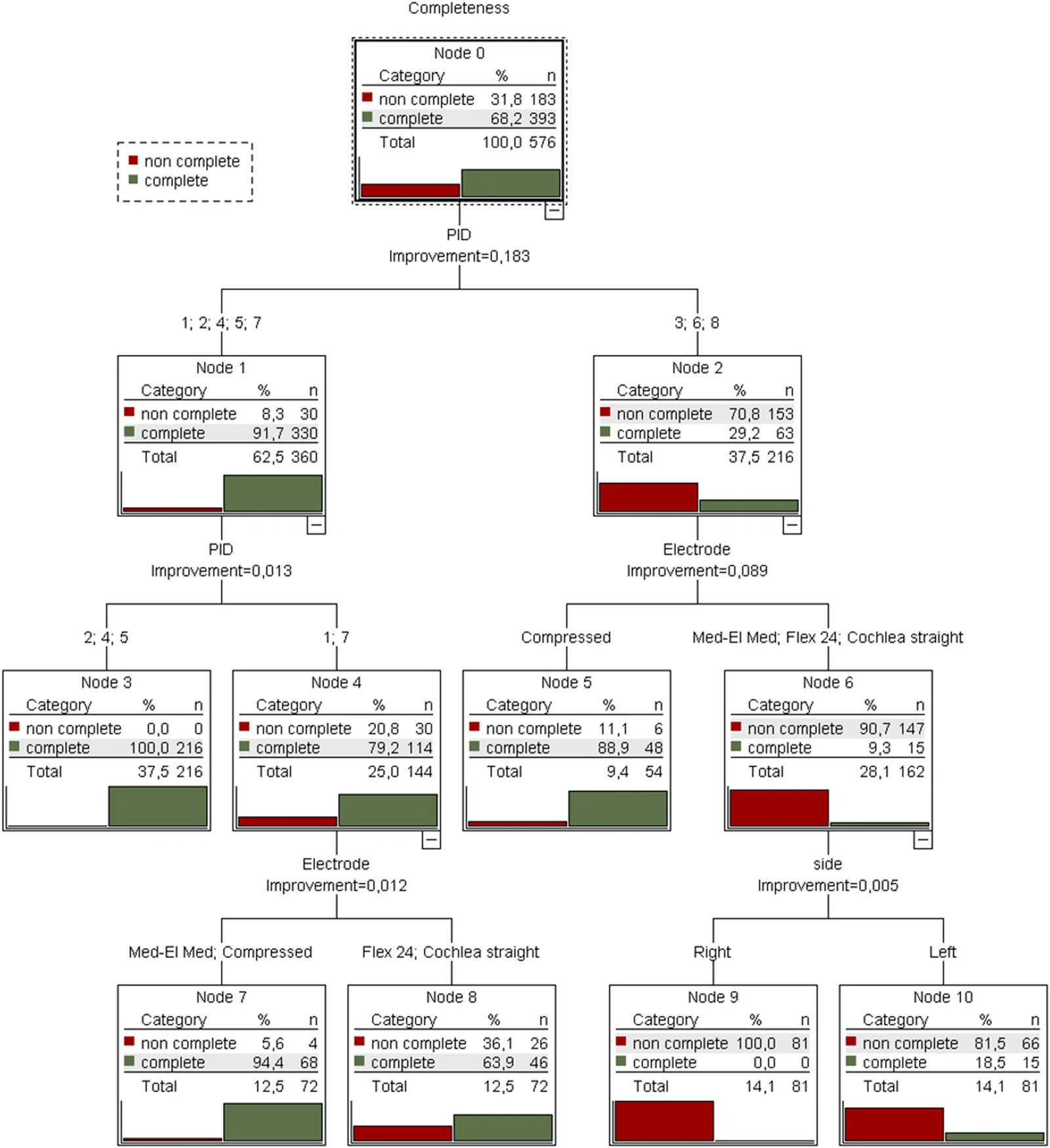
Classification tree predicting insertion outcomes based on electrode type, operator, and cochlear anatomy. The decision tree illustrates the hierarchical contribution of electrode design, operator, and patient-specific anatomy to insertion success. Each node displays the proportion of complete (green) and incomplete (purple) insertions, with percentages shown for each outcome category. Electrode type formed the earliest and strongest splits, indicating its dominant influence on insertion performance. Subsequent splits were driven primarily by patient anatomy, with favourable morphologies clustering into high-success terminal nodes and restrictive cochleae forming low-success branches. Operator identity contributed minimally to node separation. The figure demonstrates that insertion outcomes in malformed cochleae are determined chiefly by electrode stiffness and geometry in combination with underlying anatomical variation

Figure 4 presents a classification decision tree illustrating the hierarchical contribution of patient-specific anatomy, electrode design, and operator identity to insertion completeness. Each node displays the proportion of complete (green) and incomplete (purple) insertions, together with absolute case numbers and percentages. Patient identifiers (PID) are shown at intermediate nodes, enabling direct visualization of anatomy-driven outcome patterns.

At the top of the tree, the primary split is determined by patient identity (PID), separating cochleae with predominantly complete insertions from those with higher rates of incomplete insertion. This initial division highlights the dominant influence of individual cochlear anatomy on insertion outcome.

Within these patient-defined branches, subsequent splits are driven mainly by electrode type. Branches containing the MED-EL Compressed electrode are characterized by a high proportion of complete insertions, indicating robust performance across a range of anatomies. In contrast, branches associated with Cochlear™ Slim Straight and MED-EL Flex 24 electrodes show higher proportions of incomplete insertion, particularly in anatomically restrictive cochleae.

Further downstream, implantation side contributes additional stratification in selected branches, reflecting side-specific anatomical differences within individual patients. Once these splits occur, terminal nodes exhibit relatively homogeneous outcome distributions, with some nodes approaching near-complete success and others near-complete failure.

Operator identity appears infrequently as a splitting variable and does not meaningfully alter outcome distributions within nodes. Where operator-related splits occur, proportions of complete and incomplete insertion remain comparable, indicating that operator effects are minor relative to electrode design and patient-specific anatomy. For the “Ease” outcome variable, the primary split of the CRT-based classification decision tree was also determined by the patient identifier (PID). Subsequent branches were defined by electrode type, followed by implantation side, which further contributed to the separation of subgroups (see Online Resource).

## Discussion

This study evaluated electrode insertion performance in a cohort of anatomically malformed cochleae using patient-specific CT/MRI-based 3D reconstructions and 3D-printed models, comparing manual expert, manual novice, and robot-assisted insertion across four electrode designs. The key finding was that electrode design and patient-specific cochlear anatomy were the dominant determinants of insertion completeness and ease, whereas operator identity, including robotic assistance, had a limited influence. These results align with previous reports emphasizing that cochlear implantation in congenital inner ear malformations is primarily constrained by anatomical factors rather than surgical technique alone [14].

The control cochlea (Patient 4) demonstrated uniformly complete insertions across all electrodes, operators, and sides, confirming that in anatomically typical cochleae, modern electrode designs can be reliably inserted independent of operator experience or robotic assistance. This observation supports earlier findings that variability in insertion outcomes arises predominantly in malformed cochleae rather than from intrinsic limitations of the electrodes or insertion technique [8, 15].

### Electrode design as the primary determinant of insertion

Electrode design was the strongest predictor of insertion completeness and ease, the MED-EL Compressed array showed the most consistent insertion performance across patients, sides, and operators (Table 1). However, this finding should be interpreted in the context of its substantially shorter active length and intended use in anatomically constrained cochleae. In contrast, Cochlear Straight and MED-EL Flex24 electrodes demonstrated favourable performance only in less restrictive anatomies, with recurrent failures in hypoplastic or cystic cochleae. These findings reinforce the concept that mechanical compatibility between electrode array geometry and cochlear morphology is critical, particularly in malformed inner ears.

This observation is consistent with experimental and clinical literature demonstrating that electrode stiffness, diameter, and flexibility strongly influence insertion forces, scalar deviation, and achievable insertion depth [8, 9]. Dhanasingh et al. highlighted that flexible straight lateral wall electrodes are generally more tolerant of anatomical variability and reduce the risk of fold-over and scalar deviation, particularly in non-standard cochleae [15]. Similarly, micro-CT based and modelling studies have shown that cochlear size parameters correlate with scala tympani volume and insertion forces, underscoring the importance of electrode - anatomy matching [9].

From a clinical perspective, these findings have important implications for electrode selection in malformed cochleae. Although shorter or more compact electrode arrays may facilitate complete and atraumatic insertion in anatomically restricted cochleae, longer arrays may provide broader cochlear and tonotopic coverage when insertion is feasible. In conventional cochlear implant populations, longer electrode arrays have been reported to better approximate the cochlea’s natural frequency map, reduce frequency-to-place mismatch, increase spatial separation between electrodes, and improve speech perception outcomes [16, 17]. This underscores a fundamental trade-off between insertion feasibility and potential audiological benefit. In hypoplastic cochleae, individualized preoperative planning is therefore essential to balance these competing considerations rather than adopting a uniform preference for either shorter or longer electrodes.

Importantly, complete insertion should not be equated with optimal auditory performance. While shorter electrodes demonstrated superior insertion feasibility in several malformed cochleae, longer electrodes may offer functional advantages through expanded cochlear coverage and improved alignment with the cochlea’s natural tonotopic organization when successful insertion can be achieved [16, 17]. Consequently, insertion success represents only one aspect of overall implant performance and should be interpreted alongside considerations of expected auditory benefit. Future studies incorporating postoperative audiological outcomes and analyses of tonotopic coverage are needed to clarify the relationship between insertion feasibility and functional outcomes in malformed cochleae.

The present findings also support a practical framework for electrode selection in malformed cochleae. Initial electrode choice should prioritize anatomical compatibility, particularly in severely hypoplastic or anatomically restricted cochleae where complete insertion may only be achievable with shorter arrays. However, when preoperative imaging, software-assisted planning, and patient-specific modelling indicate that multiple electrode designs are anatomically feasible, electrode selection may be further refined by considering the potential advantages of greater cochlear and tonotopic coverage associated with longer arrays. In the present cohort, several cochleae demonstrated comparable insertion performance across electrode types, whereas in others longer electrodes performed similarly to shorter designs. These observations suggest that patient-specific modelling may facilitate individualized optimisation of electrode selection by balancing insertion feasibility with the potential for broader cochlear coverage, rather than prioritising mechanical insertion success alone.

### Anatomy as a hard constraint: implications of malformation severity

Patient-specific anatomy exerted a dominant influence on insertion outcomes, with strong consistency within individual cochleae across operators and sides. Favourable malformations such as IP-II and CH-III supported near-complete insertion across all electrode types, whereas severely hypoplastic or cystic cochleae consistently limited insertion depth regardless of technique. In these anatomically constrained cases, incomplete insertion likely resulted either from a mismatch between cochlear duct length and electrode length or from early mechanical obstruction related to abnormal cochlear morphology, leading to premature resistance during insertion, as previously described in malformed cochleae [18].

The observed phenotype-dependent patterns are consistent with established classifications of inner ear malformations, beginning with Jackler’s embryological framework and refined by Sennaroğlu and later morphology-based systems [5].

More recent large-cohort studies have demonstrated that malformation severity and comorbidity patterns strongly influence surgical complexity and postoperative outcomes, reinforcing the need for individualized planning in this population [14, 18].

In cochleae lacking a well-formed bony modiolus, such as IP-III, the indication for perimodiolar electrodes is particularly narrow. Prior reports recommend shorter or straight lateral wall electrodes to reduce the risk of electrode misplacement and CSF gusher in these cases [14]. Nevertheless, although modiolar-oriented electrodes were not evaluated in the present study, consideration of their potential intracochlear behaviour in malformed lumina remains clinically relevant and further emphasizes the importance of anatomy-driven electrode selection.

Although the present study focused on insertion feasibility and mechanical performance in anatomically malformed cochleae, it did not evaluate the relationship between electrode position and cochlear neural pathways. Increasing evidence suggests that patient-specific electroanatomy, particularly electrode–neural distance, substantially influences intracochlear current spread and neural activation. A study by Lei et al., demonstrated that variations in cochlear geometry and electrode positioning can significantly influence electrical field distribution, independent of insertion depth alone [19].

These considerations may be especially relevant in malformed cochleae, where distorted luminal geometry, absent or hypoplastic modioli, and atypical cochlear nerve trajectories may lead to unpredictable electrode–neural relationships despite technically successful insertions. Future studies integrating electroanatomical modelling, neural imaging, and postoperative telemetry may therefore help clarify how electrode proximity to functional neural pathways influences auditory outcomes in this challenging population.

### Robot-assisted insertion: consistency without anatomical override

Robot-assisted insertion produced outcomes comparable to those of the human operators for the endpoints assessed in this study, with the robotic system mirroring both successful and incomplete insertions within the same patient–electrode combinations. This suggests that, in malformed cochleae, insertion completeness is primarily constrained by electrode–anatomy compatibility rather than by the insertion modality alone. However, the present study did not directly measure objective robotic performance parameters, such as insertion force, trajectory precision, intracochlear pressure, scalar position, or insertion trauma. Therefore, potential advantages of robotic assistance in improving procedural consistency, reducing insertion forces, or minimizing traumatic events cannot be excluded. Previous experimental and clinical studies indicate that robotic systems may reduce insertion variability, peak insertion forces, scalar deviation, and tip fold-over by enabling stable, low-velocity electrode advancement [8, 9, 12, 13]. Nevertheless, even optimized robotic control cannot overcome the geometric limitations of a restricted cochlear lumen, emphasizing that careful electrode selection and trajectory planning remain essential in severe cochlear malformations [14, 15].

Systematic reviews of robotic-assisted cochlear implantation further indicate that while robotics may reduce traumatic events such as scalar deviation or tip fold-over, electrode choice remains a critical determinant of safety and success [8, 9]. Clinical case reports of robotic implantation in severe malformations, including IP-III, demonstrate feasibility but emphasize that careful electrode selection and trajectory planning remain essential [20].

### Role of 3D modelling and planning software

Patient-specific 3D modelling played a central role in revealing spatial constraints that are difficult to appreciate on conventional CT or MRI alone. Previous studies have demonstrated that 3D segmentation improves understanding of cochlear morphology, enables prediction of insertion depth, and supports individualized electrode selection in malformed cochleae [14]. The present findings further support the utility of physical 3D-printed models as a rehearsal and validation tool for challenging anatomies.

Incorporation of OTOPLAN^®^ into preoperative planning significantly improved sensitivity and negative predictive value compared with clinician assessment alone, reducing false-negative predictions of insertion success. This aligns with validation studies demonstrating reliable cochlear measurements and improved electrode selection accuracy using OTOPLAN^®^, particularly when compared with standard DICOM viewers [21]. However, most prior applications have focused on normal or mildly malformed cochleae, with limited systematic evaluation in severe malformations [22].

### Limitations and future directions

The present study is limited by the use of rigid 3D-printed models that do not replicate intracochlear soft tissues, fluid dynamics, or pressure-related phenomena such as CSF gusher. As a result, the models primarily reflect geometric and mechanical constraints rather than the full complexity of in vivo surgical conditions, including tissue resistance and fluid-mediated effects during electrode insertion. Imaging resolution in paediatric patients further constrains scala-level segmentation accuracy. In addition, OTOPLAN^®^ is currently limited to specific electrode families, restricting generalizability across manufacturers. A further limitation relates to the qualitative assessment of insertion difficulty. Although predefined criteria were used to standardize evaluation, classification of insertion as “easy” or “hard” remains inherently subjective and may be influenced by operator perception. Quantitative measurements, such as real-time insertion force monitoring, would provide a more objective assessment and may be particularly valuable in the context of robotic-assisted insertion. Future studies integrating force-sensing technologies could further refine evaluation of electrode–cochlea interaction.

This study is also limited by the relatively small sample size, reflecting the rarity and heterogeneity of congenital cochlear malformations, as well as the stringent imaging, modelling, and technical requirements of the study design. The cohort represents all eligible cases available within a defined time frame under consistent methodological conditions, including patient-specific 3D segmentation, physical modelling, and controlled manual and robotic insertions. Statistical methods were selected specifically to ensure valid inference in small and sparse datasets, and consistent findings across complementary analytical approaches support the robustness of the conclusions. The present work provides methodologically sound and clinically relevant insights into anatomy-driven determinants of electrode insertion in this challenging population, supporting the need for future, larger‑scale multi‑centre studies involving more heterogeneous patient cohorts.

To further improve clinical relevance, future studies should focus on patient-specific preoperative planning that integrates higher-resolution imaging, multimaterial or biomechanically realistic cochlear models, and predictive simulations of electrode-tissue interaction. Prospective clinical validation combining robotic insertion with real-time feedback such as force sensing or electrocochleography, may further reduce insertion trauma and optimise electrode positioning, thereby enhancing hearing preservation and postoperative auditory performance.

## Conclusions

In malformed cochleae, insertion completeness was influenced primarily by cochlear morphology and electrode characteristics, highlighting the importance of anatomy-driven electrode selection. Shorter electrode arrays demonstrated greater insertion feasibility in anatomically restricted cochleae, although the relationship between mechanical insertion success and functional hearing outcomes requires further investigation. Within the outcome measures assessed in this study, operator-related factors, including robotic assistance, had limited impact on insertion performance. Patient-specific 3D modelling and software-assisted planning provide valuable tools for understanding anatomical constraints and optimizing electrode selection. As robotic systems, imaging technologies, and electroanatomical modelling continue to evolve, individualized strategies tailored to cochlear morphology may further improve cochlear implantation planning and outcomes in this challenging population.

## Supplementary Information

Below is the link to the electronic supplementary material.

ESM 1 (PNG 202 KB)

ESM 2 (PNG 138 KB)

## Data Availability

The datasets generated and/or analysed during the current study are not publicly available due to patient privary and ethical considerations but may be available from the corresponding author on reasonable request, subject to applicable ethical and institutional requirements.

## Abbreviations

CH: Cochlear hypoplasia
CH-II: Type-II Cochlear hypoplasia
CT: Computed Tomography
IP: Incomplete partition
MRI: Magnetic Resonance Imaging
SNHL: Sensorineural Hearing Loss
PID: Patient Identifier

## Declarations

The authors declare that this study received in-kind support in the form of electrode arrays provided by MED-EL (Innsbruck, Austria) and Cochlear Ltd. (Sydney, Australia). The RobOtol^®^ robotic system (Collin Medical, Bagneux, France) was used for experimental insertions. The manufacturers had no role in study design, data acquisition, data analysis, interpretation of results, or manuscript preparation.

This work has not been previously published or submitted elsewhere. The corresponding author has read the author’s guidelines.
